# Heart rate variability indicates emotional value during pro-social economic laboratory decisions with large external validity

**DOI:** 10.1038/srep44471

**Published:** 2017-03-10

**Authors:** Jonas Fooken

**Affiliations:** 1 Centre for the Business and Economics of Health, University of Queensland, St Lucia QLD, Australia

## Abstract

The present study investigates the external validity of emotional value measured in economic laboratory experiments by using a physiological indicator of stress, heart rate variability (HRV). While there is ample evidence supporting the external validity of economic experiments, there is little evidence comparing the magnitude of internal levels of emotional stress during decision making with external stress. The current study addresses this gap by comparing the magnitudes of decision stress experienced in the laboratory with the stress from outside the laboratory. To quantify a large change in HRV, measures observed in the laboratory during decision-making are compared to the difference between HRV during a university exam and other mental activity for the same individuals in and outside of the laboratory. The results outside the laboratory inform about the relevance of laboratory findings in terms of their relative magnitude. Results show that psychologically induced HRV changes observed in the laboratory, particularly in connection with social preferences, correspond to large effects outside. This underscores the external validity of laboratory findings and shows the magnitude of emotional value connected to pro-social economic decisions in the laboratory.

Economists increasingly rely on insights from economic laboratory experiments, using them in theoretical research and to inform policy-makers. However, while experiments are an important research tool, economists, as experimental researchers in all branches of science, are mainly interested in what happens outside the laboratory. For this reason the external validity of laboratory results is of central importance to assess the usefulness of experiments for theory and policy-making.

Loewenstein^1^ pointed out the problem of external validity in experimental economics, which has been addressed by experimental economists in several ways. For example by connecting experiments closer to the field^2^, using laboratory experiments with field elements^3^,^4^ or field experiments on specific markets^5^,^6^. However, the quantification of external validity has been difficult, particularly when explanations for behavior observed in the laboratory are argued to be due to some (unobserved) psychological process. A prominent example for such a latent factor is the role of emotional stress that can motivate decision makers to choose in a way that would not be predicted when assuming selfish rationality.

The purpose of the current study is to provide such a quantification and to investigate the relative meaningfulness of experimental laboratory results in comparison to external or non-laboratory measures. It was hypothesized that effects in the laboratory would correspond to significant effects outside of the laboratory, being meaningful in relative terms to the outside. This relative meaningfulness or magnitude of laboratory results is referred to as *relevance* in the following. The current study provides a measurement of magnitude by using physiological data that serves as an emotional shadow value for the psychological decision making process. This shadow value provides an externally valid and quantifiable reference measure for emotional stress during decisions, hence a latent psychological process. By providing such quantification the paper contributes to the literature on the external validity of results from (economic) laboratory experiments using objective physiological data. Connecting physiological data and decisions is typical in psychology, where psychological (i.e., mainly emotional) significance is indicated by the significance of physiological reactions^7^. For example, decisions in which emotions play an important role have been connected with physiological data^8^. The current study uses heart rate variability (HRV) as such a physiological indicator that informs about the neural underlying of the decision-making process^9^. That is, HRV has been associated with activity in the prefrontal cortex, particularly in the medial prefrontal cortex (MPFC)^10^. Subregions whose activation is correlated with HRV in the context of emotional and cognitive tasks have been the (right) rostral MPFC and the left posterior putamen, respectively, the first of which has been associated with emotions and emotional physiological responses in previous research^11^.

HRV can inform about psychologically induced physiological responses of a decision maker^12^,^13^. The indicator of HRV used here, the 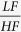 ratio, does so by informing about the sympatho-vagal balance during decision-making^12^. More information on the indicator used and on HRV is included in the Method section. HRV activity cannot be controlled deliberately and mirrors mental processes, particularly when they are emotional^14^,^15^. This makes HRV informative in the context of emotional decision-making and provides an objective shadow value in and outside of the laboratory. For this reason, the current study adds to research in *physio-economics*^16^ and *neuroeconomics*^17^,^18^,^19^, areas of research that employ neuroscientific tools to understand economic decision making. While providing interesting insights about processes in the brain and the mapping of social preferences^20^,^21^,^22^, risk and ambiguity attitudes^23^,^24^,^25^,^26^,^27^ and time preferences^28^,^29^, neuroeconomic measurement tools require moving studies increasingly into laboratory environments. This particularly accounts for brain scanning techniques, making it more difficult to determine the external validity of research findings. HRV data, in contrast, can relatively easily be collected in the laboratory and in the field, as it requires less extensive recording equipment. The current study takes advantage of this feature and uses HRV to link the laboratory to the outside. While the use of HRV as a tool for quantification of laboratory-based findings in terms of out-of-laboratory relevance is new, it is similar to other studies that measure value representations in the brain using neural markers^30^,^31^,^32^.

For comparing laboratory and field data, in a first step, the study identifies real-life events in which mental activity is exerted with differing intensity: A university exam and other mental activity that is less stressful than the exam. The difference in HRV during these two activities outside the laboratory provides with a quantitative measure of significant changes in HRV outside the laboratory. In a second step, experimental decisions for which psychological factors play a role are connected to the HRV of the decision-maker. Significant changes in HRV during experimental decisions provide a quantitative measure of HRV in the laboratory. In a last step, HRV measures paralleling experimental decisions are evaluated in comparison to measures outside of the laboratory. This comparison is possible because level differences in HRV in and outside of the laboratory are moderate and further mediated by the choice of activities outside of the laboratory used here.

To collect this HRV data the study consisted of three parts during which the HRV of participants was measured. Part 1 included a normal day (24 hours), Part 2 the day of a university exam (again 24 hours) and Part 3 an economic experiment, hence, two measurements outside the laboratory and one inside. The exam was hypothesized to be more stressful than other mental activity as measurable by HRV^33^. The first two parts are used to quantify this stressfulness outside the laboratory. Part 3 was subsequently used to determine what corresponds to a significant change in terms of HRV inside the laboratory. The three parts are outlined in Table 1. The different hypotheses for the experimental parts are further outlined below. More detail on the parts, the experimental stages and on HRV is included in the Method section.

The main hypothesis was that significant physiological changes as measured by HRV in the laboratory would be proportional to stress-induced changes in HRV outside the laboratory in terms of magnitude, underscoring the external validity of laboratory findings. Furthermore, the different stages of the experiment each tested a specific hypothesis regarding the relationship between HRV and performance in the *tasks* (stages 2,5,7) as well as choices in the *games* (stages 1a, 1b, 3a, 3b, 4). With respect to the tasks HRV indicates mental engagement. As increasing effort (hence higher mental engagement and stress) should increase performance, a positive relationship between performance and HRV was hypothesized. Stage 7 was used in addition to the two math tasks in stages 2 and 5 in order to observe if the same result would be observable in case the importance of effort was reduced (effort is more central for the math tasks).

In connection with decisions in the experimental games (stages 1a, 1b, 3a 3b, 4, more detail on the games is included in the Method section) choices may be influenced by emotional factors. That is, both pro-social decisions (stages 1a, 1b, 3a, 3b) and risk-taking (stage 4) potentially have emotional components. Pro-social decisions in the public goods game (PGG) and the dictator game (DG) may be influenced by internalized social norms about how to interact and share with others. When individuals contemplate about violating these norms during the decision-making process, they experience emotional stress. This stress motivates them to act more pro-socially (in order to reduce the stress induced by the norm violation). Dulleck *et al*.^34^ who measure HRV during tax compliance decisions, outline this argument and show a relationship between (pro-social) compliance and HRV. A similar relationship was also hypothesized here, predicting that higher HRV would be related to higher PGG contributions and DG transfers, as well as to more frequent decisions to punish selfish decisions in the PGG and the DG. An additional hypothesis was that in the punishment conditions of the PGG and the DG (stages 1b, 3b) this emotional relationship as measured by HRV would be reduced, as also individuals who do not experience emotional stress during the norm-violating decision, but make selfish choices, decide to act in accordance with the norm in order to avoid punishment.

An emotions-based connection between risk-taking and HRV was hypothesized for the betting decision (stage 4), as risk-taking has been argued to include a *feelings*-component^35^. However, there is no social norm for risk-taking, which prescribes more or less risk-taking. Therefore, the expected direction of HRV for the betting decision was not clear a priori. An effect of HRV was furthermore hypothesized when participants observed the outcome of their bet at the end of stage 4, predicting a drop in the stress level when winning the bet.

## Results

The study was designed to understand the magnitude of HRV results in the laboratory in comparison to HRV changes outside of the laboratory. In a first step main insights from the analysis outside of the laboratory are described, before looking at results from the economic experiment.

### Magnitude of HRV changes outside the laboratory

What is a significant change in HRV outside of the laboratory and what magnitude does it have? This question was investigated assuming (and the data confirms this) that the exam was more stressful within individual compared to other, primarily mental activities. An exam has been identified as stress-inducing in terms of HRV, with the HRV of students on the day of their medical exam (in the hour before taking it) being higher than HRV on a control day^33^. Table 2 illustrates the same direction of increased HRV levels comparing the exam and other daytime activities in which mental processes drive HRV changes and physical activity is minimized. Candidate activities were mental activity, computer work and communication. These were chosen as being the three most comparable activities with a sufficient number of observations (and reported by a larger number of individuals) from self-reported activities of participants during the 24-hour HRV measurement periods outside the laboratory. Examples of mental activity were ‘studying’, ‘lecture’, ‘tutorial’, ‘library’ and for communication ‘phone’, ‘phone call’ or ‘social activity’, as commented by participants in their activity protocol.

Table 2 provides a measure of magnitude for HRV changes during normal mental activity and HRV during the exam. HRV is measured by 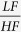 during the time of the activity. The exam is significantly more stressful (implying higher HRV) than other mental activity. As further benchmarks computer work and communication were used. Also these activities are less stressful than the exam and provide with a range between 0.27 and 0.52 units of 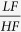 for the magnitude of HRV differences comparing exam and other mental activities.

### Magnitude of HRV inside the laboratory

#### Decisions based on pro-social preferences

##### Public good game

In the PGG participants interacted for 4 rounds (each) in two conditions, without and with punishment. Participants generally contributed high to maximum amounts and contributions increased in the presence of punishment opportunities, confirming results of the literature. Due to the difference in motivations to contribute in the PGG with and without punishment the game was analyzed separately by these two specifications.

The relationship between contributions and HRV was investigated and a significant correlation between higher contributions and higher HRV was observable in the PGG without punishment. This could be due to guilt aversion^36^, as the anticipation of feeling guilty leads to higher contributions. See Dulleck *et al*.^34^ for an elaboration of the relationship between guilt, contributions and HRV. Table 3 describes this relationship, showing higher HRV between 0.79 and 1.20 for an additional unit of contribution. This effect is large in terms of magnitude when comparing it to the difference in HRV between other mental activities and the exam in Table 2, which indicated that the exam increased HRV between 0.27 and 0.52.

Also decisions during punishment rounds were analyzed, including HRV during contributions, HRV during own punishment decisions and HRV when receiving punishment. Punishment was used in some cases, although not very frequently. It depended on the amount other players had contributed. As contribution levels were generally high, there was not much reason to punish. The analysis of contributions in the punishment part did not show a significant relationship between HRV and contributions. The direction of coefficients remained the same, but the relationship between contributions and HRV was smaller and insignificant. This finding could support the notion that moral concerns, such as guilt, trigger an emotional reaction, motivates pro-social decisions. This intrinsic moral component may partly be crowded out due to the threat of punishment. Additionally, selfish individuals who previously did not act pro-socially may be crowded in and pool with others by contributing higher amounts. However, they do not do so for moral reasons but only to avoid punishment. This could weaken the observable relationship between HRV and contributions.

Also for punishment decisions no significant relationship with HRV was observable. This insignificance could be due to low numbers of observations: Because most individuals decide to contribute in the presence of punishment, there was little reason to punish and as a result of this very few punishments of non-contributors were observable.

##### Dictator game

Linking DG transfers and HRV does not show a significant relationship, neither for the non-punishment nor for the punishment condition. While the direction, indicating a positive relationship between HRV and pro-social behavior, would be the same as in the PGG, it is not significant. This finding of non-significance in comparison to the PGG may, however, be due to a small sample of observations per individual, as participants only take two decisions per game specification in the DG compared to 4 decisions in each PGG specification.

In a next step, HRV during third-party punishment was investigated. Although the transfer they evaluate does not affect decision makers in the role of a third party, and even incur a cost when deciding to punish, punishments of low transfers were common. While receiving punishment was not correlated to HRV, a significant relationship between HRV and the decision to punish others was observable. Table 4 illustrates this result, indicating that higher HRV is correlated with a higher probability to punish. This again points to a potentially emotional role in pro-social decisions, confirming findings in the PGG. Furthermore, the magnitude of HRV during DG punishments is noticeable. Comparing the change in HRV ranging between 0.19 and 0.37 higher 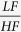 paralleling the decision to punish to the difference between the exam and other mental activities (ranging from 0.27 to 0.52) documents a large effect in the laboratory.

Together these results show that HRV changes that parallel pro-social decisions in the laboratory are substantial, corresponding to large stress-induced changes in HRV outside of the laboratory. As HRV changes in the laboratory during mental activity are influenced by emotions^12^, this particularly speaks for the importance of emotions in pro-social decisions.

#### Decisions involving effort and risk-taking

##### Math and ability tasks

Stages 2, 5 and 7 of the experiment included the two math tasks and the ability task. The tasks are comparable to the exam, intuitively as both include solving questions for which a single correct answer needs to be given. Significant within-individual correlations in HRV between the exam and the tasks are observable in the data (39% ≤ *ρ* ≤ 55%). Performance in the tasks was, however, not significantly correlated with HRV, indicating that it is more driven by underlying ability (which is uncorrelated with HRV) than by the effort (as measurable by HRV) exerted when solving tasks. There is also some indication of a positive correlation between HRV and performance in the arithmetic tasks and a negative correlation for the ability task. This could be due to the fact that the ability test is more tailored to measure unchangeable ability, while the math tasks include questions solvable less dependent on ability and more dependent on effort. However, in absence of significance this interpretation remains speculative.

##### Betting decision

In the betting decision most participants bet in a medium range and choose bets close to the average number of correct answers in the first round. The betting decision was included in order to inform about risk attitudes and risk-taking of participants. However, the data shows that the bet is strongly influenced by a projection bias: Most participants use their own performance in the first round as a reference point. For this reason the score from the first round was included as a control variable in the analysis connecting HRV and the betting decision.

Table 5 shows results from this estimation, leaving an inconclusive relationship between HRV and the bet. A potential relationship of higher HRV and higher bets can be conjectured from the data. The observed increase in HRV paralleling higher bets would again be noticeable compared to stress-induced HRV changes outside the laboratory. However, this result is less clear, reflecting the confounding factor of a projection bias and the limited robustness of significance in the result. At the same time it has to be considered that the risk measure relies on a smaller sample than the social preference measures, and hence the limited robustness in the results may be due to insufficient power and not due to the absence of a relationship.

Finally, there was no significant effect of observing the bet outcome (whether the player won or lost the bet).

In summary, the analysis of the experiment shows that contributions in the PGG without punishment were significantly related to HRV. A similar conclusion was true for third-party punishment in the DG. Hence, experimental results show that physiological states induced through emotional stress are connected to decision making in a pro-social context. Note that although in both games decisions are altruistic, their pro-social element is not the same: In the PGG contributions benefit both the decision maker and others, and may therefore also be influenced by efficiency concerns. Furthermore, in the presence of conditional cooperators, reciprocity plays a role. Third party punishment in the DG is, in contrast, purely altruistic, as there is a private cost and no personal benefit. Additionally, punishment reduces efficiency, as joint payoffs decrease.

Comparing physiological changes paralleling pro-social decisions in the laboratory with physiological changes outside the laboratory shows that effects observable in the experiment are substantial in terms of their magnitude. HRV changes in the laboratory ranged from 0.8–1.2 (PGG without punishment) and 0.2–0.4 (DG punishments) higher 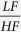. This is a large change compared to HRV changes during mental activities and an exam outside of the laboratory which ranged from 0.3–0.5 higher 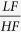.

## Discussion

Results hence indicate that physiological effects observable in the laboratory are significant in terms of their magnitude, implying a high external validity of laboratory-based results. This clearly confirms the main hypothesis of a significant magnitude of laboratory findings to the outside. This finding is particularly visible for experimental decisions that involve social preferences and moral reasoning that prescribes pro-social decisions, confirming the hypotheses regarding stages 1 and 3. Effects are reduced when social preferences and self-interested motivations jointly determine decisions, again confirming the hypothesized relationship between HRV and decisions due to intrinsic moral reasoning. Furthermore, relationships are not as clear-cut with respect to decisions that involve risk-taking. Hence, the hypothesis regarding stage 4 for a relationship between risk-taking and HRV was not supported. This might be explained by a higher emotional component of decisions that depend on social preferences, but may also be due to sample size effects. Generally, however, the findings underscore the usefulness of experiments for theoretical and applied research. They also highlight the explanatory power of latent psychological explanations in these experiments and document a substantial magnitude of emotional value observable in the laboratory. Finally, the hypothesized relationship between performance in the tasks (in stages 2, 5 and 7) and HRV was not supported by the results.

The present results have implications for the external validity of experimental findings on decision-making and underscore their relevance. HRV can be interpreted to reflect psychologically induced physiological stress during the decision-making process. However, arguably, the experiment did not directly measure subjective emotional states, which limits the reading of the result as induced by emotions because it relies on the interpretation that HRV is induced by emotions. Accepting this interpretation, as would be reasonable based on the literature on the connection of emotions and HRV during mental activity^14^,^15^, the results indicate the importance of emotional factors in the context of pro-social decisions. Even more, the results highlight the external validity of findings from laboratory experiments in non-laboratory contexts. As such they add to a literature comparing decision-making in and outside the laboratory^2^ that cannot assess latent components of decision-making, such as emotional value. Assessing latent processes in the brain during decision making is typically done using brain scans, which makes external validity testing more difficult. A number of research studies has shown links between HRV and brain regions in the context of emotions, including the anterior cingulated cortex, anterior insula and dorsolateral prefrontal cortex^37^,^38^. These regions have also been shown to be activated during experimental economic decisions^39^,^40^. Together with other research that shows the connection between neural activation in the MPFC and HRV during emotion^9^,^10^,^11^, this indicates an important and externally valid emotional component in (pro-social) economic decisions. The findings of the current study measure this emotional value and document that experimental results have a noticeable correspondence in terms of magnitude to stress in reality. This finding strengthens the role of experiments as a tool to inform theory and policy-makers. That is, laboratory findings on economic decision-making are relevant outside the laboratory and are informative when drawing conclusions about decision-making based on laboratory findings. In particular, this result accounts for underlying (unobservable) psychological factors that play a role in decisions, particularly emotions.

Finally, the study also tried to compare the magnitude of different types of preferences measured in the laboratory. This provides a contribution to research investigating (latent) value representations during different types of decisions in the laboratory, such as pro-social decisions or risk-taking^30^,^31^,^32^. While previous studies measured these values only in the laboratory, the current study shows that very similar activation as in laboratory-based decision-making is observable outside the laboratory. Comparing the different preference types shows that social preferences are linked to HRV. In comparison, the connection was less clear for risk-taking in a betting decision, although the literature would suggest that also risk-taking has an emotional component^35^ which would be measurable by HRV^41^. While the results from the current study do not reject an important role of emotions in risk-taking decisions, they show that the clearer results with regard to emotional factors are visible for social decisions. However, the reason for less robustness of the result regarding risk attitudes could also be the lower sample of risk-taking decisions. Hence, results do not dismiss a connection between HRV and risk-taking or the external validity of risk measures, but are inconclusive. Therefore, a comparison between the emotional value of different preferences elicited in the laboratory is better addressed in future research which focuses on results in the laboratory only.

## Materials and Methods

### Study design and data collection

Data was collected over 2.5 years from 2010 to 2012 with mostly first-year undergraduate students as participants. Ethics approval was provided by the QUT University Human Research Ethics Committee (ethicscontact@qut.edu.au) before the start of the study and all participants provided their written informed consent before the start of any measurement. The study was carried out in accordance with this approval and the resulting guidelines. Participants were recruited as volunteers via ORSEE^42^ and in an open call before a lecture. 55 participants (49% female, average age 21 years) completed all parts of the study. Additionally, 2 participants took part in the experiment, but were not measured during the exam. As for 1 participant decision times during the experiment were not recorded (hence experimental data from this participant cannot be used), this results in samples of 56 participants for experimental decisions and between 28 and 54 participants for decisions outside the laboratory. Lower samples occurred on some daytime activities, such as computer work, which has not been recorded during the 24-hour measurements by every participant. Activities were also sometimes recorded more than once by some participants, leading to larger numbers of observations that can be included in the analysis.

The number of observations included in the analysis of experimental decisions depends on the game (some games are repeated more often than others and consequently include more observations) and may be reduced by (a small number of) data measurement errors.

As common in economic experiments, participants received payments for their participation, which were 50 Australian dollars (fixed amount) for the two 24-hour measurements and on average 24 Australian dollars depending on their decisions and performance in the experiment.

#### 24-hour measurements

For 24-hour measurements participants arranged an individual time via email with the experimenter to start the HRV recording. On their first appointment they received information about the study, were given an ethics consent form, were explained how to attach the heart rate monitor and how to handle it during the measurement period. They received a heart rate monitor, attached 3 electrodes to their chest (in private), connected the monitor to the electrodes and started the measurement. The quality of the recording was tested with an infrared connector to the heart rate monitor. Participants received an activity protocol to be filled out during the day, recording major activities (such as studying, walking, eating, sleeping, watching TV, etc.) with its corresponding time.

Participants were also told that a minimum time interval useful for the analysis would be 5 minutes. Technically HRV recording devices allow for much shorter time intervals and shorter time intervals were used in case they were recorded as such by participants. This guideline was rather chosen to ensure that there was little interference of participant’s daily routine.

The normal day measurement was done before the exam day measurement, in order to familiarize participants with the recording device and to not induce additional stress on the exam day. Otherwise instructions were the same for both measurements. On the exam day participants were required to wear the monitor in a 24-hour period, which had to include the exam and one additional hour after the exam. There were also requirements for the exam. It had to last between 1 and 2 hours (including reading time) and to count between 40% and 60% of the final grade. Exams typically were undergraduate exams in economics or business.

#### Economic experiment

Each participant’s HRV was also recorded during the economic experiment. The experiment was implemented in a computer laboratory using z-Tree^43^. After the completion of the two 24-hour measurements, participants were contacted via e-mail and experimental sessions were scheduled with multiples of 3 participants in one session. This small number was necessary for coordinating participants. However, it also limited the number of possible rounds with rematching in stage 3. A number of different games and tasks were included to assess the relevance, as indicated by HRV, of elicited preferences over uncertain and social outcomes. The games and tasks were presented in 7 stages as outlined in Table 1. Of these, the first 6 were payment-relevant as typical in economic experiments. In the 7^*th*^ stage participants were only informed about their score, but not paid based on their performance. Furthermore, in the first 6 stages instructions were read out aloud by the experimenter, while in the last stage participants were asked to read instructions privately. (Stages 2 and 5 had identical instructions; this was pointed out to participants in stage 5 while instructions were not repeated in full.) The first 6 stages were treated differently from the 7^*th*^ representing experimental standards in economics and psychological research. Experimental payoffs of the first 6 stages were calculated in experimental dollars that paid 20 experimental dollars = 1 Australian dollar, a rate announced in the beginning of the experiment. The experimental stages are summarized below (full instructions are included in the Supplementary material). Stages 1 and 3 were chosen to elicit social preferences with varying elements of (potentially emotional) moral reasoning and stage 4 to elicit (also potentially emotional) risk taking, as emotional factors in decisions are important when connecting them to HRV. The other stages were aimed to record (stressful) effort.

#### Stage 1: Public good game

In stage 1 participants played a standard public good game (PGG), which has extensively been studied in experimental economics^44^. In the PGG participants are provided with an endowment (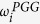 = 10) which can be kept for private use or contributed in full or partly (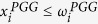) to a common account (the *public good*). The sum of all contributions to the common account of a group (of *n* = 3 players) is increased by a factor (2) and equally split between the group members 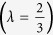. Each individual hence receives 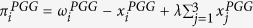. Contributions are more costly than the benefit received from own contribution. Selfish payoff-maximizing players will therefore contribute nothing. However, typically positive contributions, at least initially, can be observed for most players and are interpreted in the context of pro-social decisions, with contributions indicating willingness to sacrifice own resources for a common benefit. The PGG with these specifications was played for 4 rounds.

Afterwards a modification of the game was played. Participants, after all group members had made their contributions, were able to observe which player had contributed which amount. They were then able to punish other players. Punishing was costly (*cost*_*ij*_ = 2 per punished player *j*) and punished players had to pay a fine (*fine*_*ji*_ = 4 for every player *j* that punished them). Payoffs hence change to 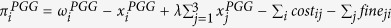. The presence of punishment opportunities typically increases contribution levels and often punishment is exerted towards (remaining) non-contributors^45^,^46^,^47^. This second variant of the PGG was again repeated for 4 rounds.

#### Stage 2: Math task part 1

In stage 2 participants were asked to solve 20 arithmetic questions in 10 minutes without pen and paper. Half were cross-sums (345 would be 3 + 4 + 5 = 12) and half were cross-multiplications (345 would be 3*4*5 = 60). Cross-sums and cross-multiplications randomly changed between periods. The two types of tasks (instead of using just cross-sums) were used so that participants needed to check the task and focus again for each question. The difficulty was increasing over the periods. For every question correctly answered, participants received 8 experimental dollars.

#### Stage 3: Dictator game

Stage 3 included the so-called dictator game (DG). See Engel^48^ for a literature review and a meta-analysis of results. In this game two players are matched, one transferring player (the *dictator*) and one recipient. The dictator can transfer any fraction of her endowment (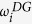 = 10) to the recipient. The recipient receives this amount but cannot communicate or transfer anything back. This will lead selfish dictators to transfer nothing. Positive transfers (common are values up to half of 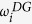) are usually interpreted as pro-social preferences or conformity to fairness norms. The game with these parameters was played for 2 rounds.

The DG was then modified. After the dictator had decided, a third player, unaffected by the transaction, had the possibility to evaluate the transfer and punish the dictator. A *fine* (of 4) was imposed on the punished player. Punishing had a *cost* (of 2). This modification was played for 2 rounds.

#### Stages 4 and 5: Betting game and math task part 2

In stage 4 participants were informed that they would again solve questions as in stage 2. They were also provided with 30 experimental dollars to bet on the performance of another, randomly assigned player in this second round of questions. The bet was on how many answers this player would (at least) answer correctly. Participants were informed about the average performance of all participants in the first round of question solving, but no information on the assigned player was provided. Table 6 provides the betting odds. Participants submitted their bet and then started the second task with the same structure as in stage 2. At the end participants were informed about their performance and on the outcome of their bet.

#### Stage 6: Bidding game

In stage 6 participants played a bidding game not further described and analyzed here. The reason for this is that the game has no clear behavioral prediction (i.e., there is no pure strategy equilibrium and a mixed strategy cannot be identified in the data; more information and an analysis of this stage is available upon request).

#### Stage 7: Ability test

In stage 7 participants performed an ability task that was similar to an cognitve skill test, giving them 12 minutes to solve as many out of 50 questions as possible. The difficulty of the questions increased when advancing further. Three types of questions were used (of about equal proportion), one testing numerical reasoning (e.g., ‘What number should come next: 27 9 3 1 1/3 1/9 1/?’), the second verbal reasoning (e.g., ‘FREQUENT is the opposite of 1 regular, 2 multiple, 3 rare, 4 loud, 5 ever’) and the last spatial reasoning (e.g., indicating which from geometrical figure can be created by two triangles, given a set of 5 choice options).

### Heart rate variability measurement and interpretation

HRV was used to assess the physiological state of participants. To measure HRV, portable Electrocardiogram (ECG) Holter Recorders (Medilog AR4) were used with 3 electrodes attached to a participant’s chest. Following the recommendation of the supplier, the points of attachment were (i) under the right collarbone, (ii) 3 fingers under the right breast and (iii) two fingers under the left breast of the participant. From the recorded ECG the heart rate variability for a given period can be calculated. Here heart rates during the measurement period are used to determine HRV and HRV averaged over decision or activity time.

HRV describes changes in the heart rate over time. As a physiological indicator it is mainly used in medical research^49^ and has been linked to psychological, emotional and mental states^13^. The understanding of HRV rests on its connection to the autonomous nervous system (ANS). The ANS is influenced by the sympathetic and parasympathetic systems whose relative influence is mirrored in the 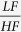 indicator. The sympathetic system is responsible for fight-or-flight responses, using sympathetic nerves and hormones (particularly adrenaline). The parasympathetic system controls rest and relaxation through pacemaker cells. The relative influence of the sympathetic and parasympathetic system in the ANS is reflected in the heart rate. While both systems are constantly active, the degree to which one of the systems controls the heart rate in a given period varies. The relative influence of the sympathetic to the parasympathetic system can be determined based on changes in the heart rate because the two systems operate at different speeds. Changes in the heart rate due to increased sympathetic activity have a longer time horizon compared to parasympathetic activity, allowing for a decomposition of the heart rate into different frequencies, which reflect the importance of sympathetic and parasympathetic activity. Higher activation of the low frequency (LF,.04-.15 Hz) corresponds to higher sympathetic activity; higher activation of the high frequency (HF,.15-.4 Hz) corresponds to higher parasympathetic activity. More detail on the decomposition using frequencies is described in the Supplementary material. The 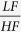 ratio is also referred to as an indicator of the sympatho-vagal balance. This indicator is used here instead of the LF alone, as it has been argued that the LF may be influenced by parasympathetic activity and the ratio has been used to solve this issue^12^. See the Supplementary material for more illustrations and definitions of the indicators used.


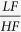
 serves as an indicator of psychologically induced physiological stress^12^ and conveys information about psychological states^50^; for example, a higher ratio of sympathetic to parasympathetic activity has been connected to increased mental stress^51^. Due to its reflection of the sympatho-vagal balance, HRV entails an interpretation, which goes beyond being a correlate of mental activity. As higher HRV reflects increased sympathetic or less parasympathetic activity it indicates higher stress. This stress is understood in relative terms within individuals and makes within-subject analysis advisable. Making within-subject comparisons is also necessary considering that some participants might be absolutely more or less stressed or react stronger to any stressor, while showing only small changes in behavior^52^. Hence, changes in HRV may not have the same magnitude of effects across individuals and individual heterogeneity should be taken into account in the analysis.

Interpretations of HRV and its connection to economic decisions, as investigated here, are based on the concept of measuring (mental) stress, which is also reflected in the existing literature using HRV data in economics research^34^,^41^,^53^,^54^,^55^,^56^. More particularly, stress in connection to decisions and reflected in HRV indicates mental stress induced by emotions^12^. The results with respect to the magnitude of changes in HRV in and outside the laboratory are therefore best understood as reflecting mental stress. In the laboratory this stress may be due to different sources of mental stress depending on the experimental elements. While HRV outside of the laboratory is typically also influenced by physical activity, this factor is controlled (i.e., not present or at least minimized) in the laboratory. For the activities outside the laboratory used for comparison here physical activity is also minimized. Analysis of the data shows that the level of HRV is similar for measurements in and outside the laboratory for those activities used for comparison. Therefore, physiological changes as measured by HRV are induced by mental activity both for activities in the laboratory and for those activities outside the laboratory used here.

### Analysis of data

For measures outside the laboratory HRV during activities of the 24-hour period was determined as a series of data points with 6 seconds length. Hence, in the current study HRV is not accoring to the 1996 Task Force standard^57^,^58^. These 6 second data point were then averaged over the duration of an activity. The most common daytime activities were considered. Candidate activities were the exam, mental activity, computer work, sleeping, watching TV, walking or cycling, resting or relaxing, eating and drinking, communication, using public transport and driving. The analysis below uses on the exam (average duration 66 minutes, s.d. = 19), mental activity (av. dur. 83 min., s.d. = 47), computer work (av. dur. 78 min., s.d. = 50) and communication (av. dur. 47 min., s.d. = 39). The reason for this was to use activities outside the laboratory, which are comparable in terms of HRV levels to the experiment. For example, overviews show that during walking and cycling (sleeping) HRV is on a higher (lower) level than HRV during the experiment and therefore not as useful for a reference point. Table 7 shows average levels of HRV for the most common activities as well as the experiment. The level of HRV during the experiment (which was done in a computer laboratory) is similar to other computer work outside the laboratory, however, with a higher variance. Generally, for all of the 4 activities chosen for comparison to the experiment the absolute level of HRV activity is similar to the level in the experiment (high within-subject correlations of.53 ≤ *ρ* ≤ 0.82 and a having a similar average, compared with other states, such as walking). Furthermore, these activities are sedentary (like the experiment) and HRV during these activities is (as HRV during the experiment) driven by mental processes.

For analyzing HRV in the laboratory, experimental decisions were merged with HRV data. HRV recorded at the moment of a decision was identified, defined as an interval of 10 seconds on each side of an experimental event. Every event in the experiment, such as entering a screen or clicking a button is recorded with its corresponding time. Events were matched to HRV data such that the event time served as the midpoint of a 20 second interval. Within this interval HRV, which was recalculated new for every 6 seconds, was averaged over the 20-second interval. The analysis below used the moment of clicking the OK button (which finalized the respective decision) as the event to be linked to the decision. Alternative analysis to test the robustness of results used the average HRV during a decision, from entering the decision screen to leaving it. There were no qualitative changes to the results.

The result section describes in more detail the experimental findings in which a significant within-individual relationship between HRV and decisions was visible. Other results, hence those not showing significant results in the other games or the three tasks, are only mentioned briefly.

The analysis of and comparison across other daytime activities showed a strong individual component in HRV, blurring cross-individual analysis if disregarded. That is, individual heterogeneity can have an effect almost as strong as differences between physical and mental activities. For this reason individual heterogeneity was taken into account by using fixed-effects regression models.

## Additional Information

**How to cite this article:** Fooken, J. Heart rate variability indicates emotional value during pro-social economic laboratory decisions with large external validity. *Sci. Rep.* **7**, 44471; doi: 10.1038/srep44471 (2017).

**Publisher's note:** Springer Nature remains neutral with regard to jurisdictional claims in published maps and institutional affiliations.27-Feb-2017 17:03

## Supplementary Material

Supplementary Material

## Figures and Tables

**Table 1:** Elemtents of the study.

**1 - Normal day measurement of HRV**	
	24-hour recording together with activity protocol (filled by participants)
**2 - Exam day measurement of HRV**	
	24-hour recording together with activity protocol (filled by participants); the 24-hour period included a university exam of 1–2 hours
**3 - Experiment measurement of HRV and experimental decisions**	
*Stage*	
1a	Public good game (PGG) without punishment (4 rounds)
1b	Public good game with punishment (4 rounds)
2	Math task 1 (solving cross-sums and -multiplications for 10 minutes)
3a	Dictator game (DG) without punishment (2 rounds)
3b	Dictator game with third-party punishment (2 rounds)
4	Bet on performance of another player in a second math task (with information about average first round performance)
5	Math task 2 (solving cross-sums and -multiplications for 10 minutes) and outcome of the bet
6	Bidding game (excluded from analysis, see Method section)
7	Ability test (similar to cognitive skill test)

**Table 2:** HRV differences between the exam and other activities.

	Exam - mental activity	Exam - computer work	Exam - communication
Exam- 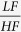	0.45**	0.27^†^	0.52**
	(0.17)	(0.16)	(0.19)
Individual Effects	Yes	Yes	Yes
Number of Individuals	56	46	28
N	343	208	145

Note: Results from fixed-effects regressions of the exam on HRV for activities during the 24-hour period. Standard errors (clustered by individuals) are in brackets. **p < 0.01, *p < 0.05, ^†^p < 0.1.

**Table 3:** Relationship between contributions and HRV in the PGG without punishment.

	PGG1	PGG2	PGG3
Decision- 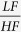	1.20*	1.16**	0.79^†^
	(0.60)	(0.40)	(0.43)
Individual Effects	Yes	Yes	Yes
N	224	208	224

Note: Results of fixed-effects regressions of HRV during the contribution decision on the level of contributions. The average HRV across all participants for the corresponding decisions was 1.79. Specifications PGG1-3 use alternative measures of HRV 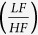. PGG1 uses the direct measure at the moment of decision making, PGG2 and PGG3 normalize this value by the average individual 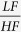 during the experiment and the round, respectively. Standard errors (clustered by individuals) are in brackets. **p < 0.01, * p < 0.05, ^†^p < 0.1.

**Table 4:** Relationship between 3^*rd*^ party punishments and HRV in the DG.

	DG1	DG2	DG3
Decision- 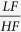	0.37*	0.19^†^	0.29*
	(0.15)	(0.10)	(0.13)
Individual Effects	Yes	Yes	Yes
N	111	103	112

Note: Results of fixed-effects regressions of HRV during the punishment decision on the (binary) decision to punish. The average HRV across all participants for the corresponding decisions was 2.58. Specifications DG1-3 use alternative measures HRV 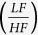. DG1 uses the direct measure at the moment of decision making, DG2 and DG3 normalize this value by the average individual 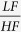 during the experiment and the round, respectively. Standard errors (clustered by individuals) are in brackets. **p < 0.01, * p < 0.05, ^†^p < 0.1.

**Table 5:** Relationship between the betting decision and HRV.

	BET1	BET2	BET3
Math points part A	0.23*	0.21*	0.23*
	(0.11)	(0.10)	(0.11)
Decision- 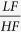	0.24	0.39*	0.28
	(0.19)	(0.19)	(0.19)
Individual Effects	Yes	Yes	Yes
N	56	52	56

Note: Results of fixed-effects regressions of HRV during the betting decision and own score in the first round on the amount of correct answers by the assigned player. The average HRV across all participants for the corresponding decisions was 2.37. Specifications BET1-3 use alternative measures of HRV 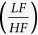. BET1 uses the direct measure at the moment of decision making, BET2 and BET3 normalize this value by the average individual 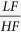 during the experiment and the round, respectively. Standard errors (clustered by individuals) are in brackets. **p < 0.01, *p < 0.05, ^†^p < 0.1.

**Table 6:** Table of betting odds for participants.

Number of correct answers	4	8	10	12	14	16	18	20
Factor multiplied with 30 in case of winning	1.2	1.5	2	2.5	3	4	6	10

**Table 7:** Average HRV during most common activities.

	Bus, metro, ect.	Walking, cycling	Exam	Mental activity	Communication	Experiment	Computer work	Sleep	TV
Mean	2.51	2.43	2.36	2.31	2.27	2.15	2.14	2.14	2.12
St. Dev.	1.82	1.48	1.53	1.29	0.95	1.50	1.12	3.11	1.76
Data points	129	230	52	292	94	18648	159	102	109
Participants reporting activity	44	52	47	54	28	56	46	53	45

Note: The table shows the average and standard deviation of HRV for the most common activities as reported by participants during the 24-hour measurements, as well as for the experiment. Decisions are ordered by average HRV. The table also includes the number of data points used for calculating the average HRV and the number of the number of participants reporting an activity. More than one data point can be reported by a participant during the 24-hour activities in case the participant did an activity more than once. The high number of data points for the experiment is due to the fact that all experimental events, such as entering or leaving a stage, or making a decision provides a data point that was included when calculating the average HRV over the entire experiment. The number of data points used in the experimental analysis is lower because only data points during decisions of interest were considered in connection with these decisions.
