# Supplementary material for: Heart rate variability indicates emotional value during pro-social economic laboratory decisions with large external validity
Source: Sci Rep. 2017 Mar 10;7:44471. doi: 10.1038/srep44471 (PMC5345012; doi:10.1038/srep44471)
Supplement: Supplementary Material [file srep44471-s1.pdf]

SUPPLEMENTARY MATERIAL to
Heart rate variability indicates emotional value during pro-social economic laboratory decisions with large external validity

Jonas Fooken

Experimental instructions

The following sections document the instructions of the different stages of the game (section titles were not provided to participants)

Public good game (PGG)

General introduction

Instructions
You are about to begin the economic experiment. Before the experimental session starts, please read the following instructions carefully. This is important because the final payment you receive for participating in the experiment can be affected by how well you understand the instructions and your decisions during the game.
Please note that all information provided during the experiment is treated confidentially.
You are prohibited from communicating with other participants during the experiment.
All the decisions you make during the game are anonymous in the sense that other participants cannot attribute decisions to other individuals.
If you have any questions now, or during this experiment, please indicate this by raising your hand. Do not talk to other participants

The experiment consists of several games and task solving exercises. A game in this context means that players make decisions that directly or indirectly affect the outcomes of other players. Those games are played for a (small) number of consecutive rounds, during which the same game will be repeated. At the beginning of each game, you will be informed about the number of rounds played. The task solving exercises will always be played for only one round.
The dollar values you see on the computer screens are 'experimental dollars'. Your income and payoffs are calculated using these experimental dollars. Your decisions and answers hence determine how much you will receive for participating in the experiment. The conversion rate from experimental dollars to Australian dollars is:
100 experimental dollars = 5 Australian dollars.

PGG without punishment

Instructions

In the following game you are supplied with 10 experimental dollars at the beginning of each round. You can use this amount to either keep it to yourself or to contribute up to 10 experimental dollars to a common pool.

The common pool is made up of the contributions from 3 players and jointly owned by them. All players will remain in their group for all rounds of this game. A group consists of three people.

After all group members have made their contributions to the pool, the total contributions will be multiplied by the factor 2. Subsequently the pool will be equally split between the group members. I.e. the split is such that each member of the pool receives an equal amount. This also means that the redistribution will be independent of what a player previously contributed to the common pool.

All decisions made in the following rounds, both yours and those of others will be anonymous; that is, you will be unable to observe which player has contributed which amount to the common pool.

The game will be played for 4 rounds.

Finally, please note that contributions need to be multiples of one experimental dollar.

PGG with punishment

Instructions modification

For the next 4 rounds the game will continue to be played as before. I.e. you can make contributions to a common pool, which are then multiplied by the factor 2 and equally split between the members of the group.

However, contrary to before, it will be possible to determine which player has contributed what amount. Hence, in an additional step, all members of a group will be informed about the other players' contributions.

Having received this information, group members can then decide if they want to punish other players for their contributions. If a player is punished, it will mean that he has to pay a "fine" of 4 experimental dollars, which will be subtracted from his final payoff.

The decision to punish other players will, however, be costly for the player who punishes others. For every punishment made, the punishing player will incur a cost of 2 experimental dollars.

It will not be possible to determine which player has punished other players. That is, the punishment is anonymous.

Arithmetic questions pt.1

Instructions
In the following section you will be asked to solve two different types of mathematical questions. The questions will ask you to either solve the sum of the digits provided, or the product of the digits provided.
To solve the questions you have to make the following calculations to find the solution:
For the sum of digits, add up all the digits. For example, to solve 25901, arrive at the solution by calculating 2+5+9+0+1 = 17.
For the product of digits, multiply the digits. For example, to solve 3172, arrive at the solution by calculating 3*1*7*2 = 42.
The difficulty of the questions will increase with when proceeding in the game. Please also note that questions for which you will have to calculate the sum of digits and questions for which you have to calculate the product of digits will randomly change between periods. Hence, make sure you check the task at the beginning of each question.
Each correct answer is worth 8 experimental dollars. There is a time-limit of 10 minutes for all questions (on average 30 seconds per question).

Dictator game (DG)

DG without punishment

Instructions
For the following rounds, a new game will be played. Please go through the instructions carefully again.
In this game, players are assigned the role of the transferring player or the role of the recipient.
The transferring player is supplied with 10 experimental dollars. The transferring player can then either keep this amount or transfer up to 10 experimental dollars a second player who has been randomly matched in the role of the recipient. The recipient will receive the transfer, but will not have the opportunity to communicate anything back to the transferring player.

The game will be repeated for 2 rounds.
Please note that all transfers need to be multiples of one experimental dollar.

DG with punishment

Instructions modification
In the next rounds, the game will again be slightly altered. Again, there will be a transferring player and a recipient. But additionally, after the decisions of the transferring players have been made, the amount of their transfers will be made available to a third player, who is unaffected by the transfer decision. That is, this player is neither a transferrer nor a recipient in the transaction.
The third player can then evaluate if the transfer has been appropriate. If the evaluating player thinks that the transfer has not been appropriate, the third player can impose a fine on the transferring player of 4 experimental dollars. However, for imposing the fine, the third (punishing) player will have to pay 2 experimental dollars.
Again, the game will be repeated for 2 rounds.

Betting game

Instructions
In the following section, all players will solve test questions that are similar to those solved earlier in the experiment. As before, for every question correctly answered, they will receive 8 experimental dollars.
Additionally to solving questions yourself, you have randomly been assigned another player on whose performance you can bet. Similarly, another player will be able to bet on your performance. The table below provides you with information on the betting odds. There is only one fixed amount of 30 experimental dollars, which you can place as a bet.
The number of correct answers on which you place your bet is a minimum number; that is, when your assigned player answers more questions correctly than you have chosen, you will still win your bet. If the number of correctly answered questions is below your bet, you will lose. The odds on your bet increase with the number of questions that you bet your assigned player will answer correctly.
Example: If you bet that the player that has been assigned to you will answer 10 questions correctly, you will receive 2*30 experimental dollars if

your assigned player answers 10 or more questions correctly and will lose your bet of 30 if your assigned player answers less than 10 questions correctly.
Please also note for your further information:
The average number of questions correctly answered in the first round was .
Please tick the number of questions that you think will be answered correctly on the lower right to make your bet.
Table 1 lists the corresponding factors of multiplication for the correct number of answers in the bet.

Table 1: Table of betting odds for participants

Number of correct answers	Factor multiplied with 30 in case of winning
4	1.2
8	1.5
10	2
12	2.5
14	3
16	4
18	6
20	10

Arithmetic questions pt.2

Instructions

In the following section you will again be asked to solve two different types of mathematical questions, i.e. to either solve the sum of the digits provided, or the product of the digits provided. To solve the questions you have to make the following calculations to find the solution:
For the sum of digits, add up all the digits. For example, to solve 25901, arrive at the solution by calculating 2+5+9+0+1 = 17.
For the product of digits, multiply the digits. For example, to solve 3172, arrive at the solution by calculating 3*1*7*2 = 42.
The difficulty of the questions will increase with when proceeding in the game. Please also note that questions for which you will have to calculate the sum of digits and questions for which you have to calculate the product

of digits will randomly change between periods. Hence, make sure you check the task at the beginning of each question.
Each correct answer is worth 8 experimental dollars. As pointed out before, furthermore a randomly chosen second player has been able to place a bet on your performance and will win or lose depending on your performance.
Again, there is a time-limit of 10 minutes for all questions (on average 30 seconds per question).

Bidding game

Instructions

For the next 4 rounds the following game will be played: At the beginning of each round, all participants will be provided with 10 experimental dollars that they use for bidding for a common pot. Bids can be made between 0 and 10 experimental dollars. All bids will directly be paid into the pot.
Remaining money out of 10 that has not been used for bidding in a period will be paid to the player at the end of the round.
The size of the pot will be made up of all bids that have been submitted by all players plus an additional amount of 10 experimental dollars, which has been placed in the pot by the experimenter. After all players have placed their bids, the pot will be distributed between the players as follows:

- The player with the highest bid will be paid one half of the money in the pot.
- The player with the second highest bid will be paid one third of the money in the pot.
- The player with the lowest bid will be paid one sixth (the rest) of the money in the pot.
- In case two or three players submit an equal bid, they will equally share the amount that they would have won together. For example, if the highest bid is shared by two players, then each of them will receive one half of (one half + one third), i.e. 5/12.

Ability test

Now you will be asked various types of questions that must be completed without a calculator or any other aid.

Directions (Please Read Carefully)
This test contains 50 questions that increase in difficulty. It is unlikely that you will finish all of them, but do your best.
After you start you will have 12 minutes to provide as many correct answers as you can. Work carefully but do not spend too much time on any one question. You can skip questions but you cannot return to unanswered questions on previous screens. Be sure to write your answers in the fields provided. Before you begin taking this test, please answer the Sample Questions below.

HRV Measurement Equipment and Data processing

When interpreting heart rate variability (HRV) as an indicator of mental stress in the context of behaviour it is necessary to identify whether the sympathetic or the parasympathetic system are more active in a particular time interval. This is so because the underlying intuition of HRV rests on interpreting the activity of the sympathetic and parasympathetic systems as reflecting physiological and mental processes within the body [1,2]. Furthermore, and more short-cut, HRV measures are also correlates of activities in certain brain regions [3,4] and parallels mental activity in the brain.

The use of HRV measures builds on the observation that heart beats are not independent events, but a realization of a continuous charging and releasing process of electrical potentials. This continuous process is the heart rate signal. The electrocardiogram (ECG) recording allows to use information on the strength of a heart beat based on the length of the so called QRS complex to construct the signal (see Figure 1 for a schematic illustration of the QRS complex). Subsequently, consecutive QRS data points are transformed into a continuous measure.

Figure 1: The QRS complex in a typical heart rate signal

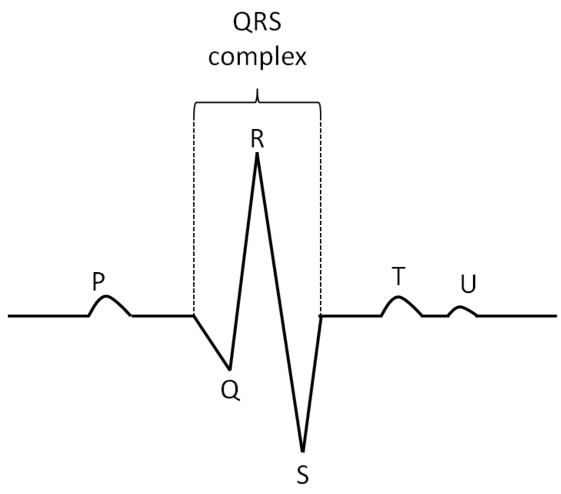

Here HRV is represented as a power spectral density (PSD). This HRV estimation process builds on the fact that the heart rate signal can be ex-

plained using a decomposition into time-frequency distributions.[1] The PSD assigns a power value to each frequency or wave length for each point in time (more exactly for each time interval). The heart rate signal is thus represented in terms of importance (power) of a particular wave (frequency) at a point in time. The PSD can be estimated using different techniques. Results presented in this paper are based on the Smoothed Pseudo Wigner-Ville Distribution method (SPWVD, a wavelet transformation) [5]. This method is desirable here as it allows for a high time-frequency resolution and is, compared to other methods, best suited to detect isolated, short and non-periodic changes in the heart rate. Additionally, the results presented in this paper were scrutinized using two other widely used methods, the Short-term Fast Fourier Transformation (SFFT) and the Autoregressive Spectral Estimation (AR) [6], providing the same qualitative results.

Data Collection and QRS detection

The heart rate measurement equipment used was the non-intrusive Holter Medilog ECG Recorder AR12. This monitoring device has a pocket-size (10x7x2.5cm) and is attached to the chest of the human subject using three conducting electrodes. To reduce the potential of irregularities and noisy readings, the heart rate monitor function was tested using the IrDA (Infrared Data Association) interface before the beginning of each recording session. The monitoring device records the ECG with a high sampling rate of 4096 Hz. Furthermore, it records respiration levels and has a build-in QRS detection algorithm. The data recorded is stored on an CF disk and can be exported into Matlab. From this data the HRV was determined using a user-written program.

References

[1] Appelhans BM, Luecken LJ. Heart rate variability as an index of regulated emotional responding. Review of General Psychology. 2006;10(3):229.

[1]For example a composition of shorter and longer sine and cosine waves; for a more intuitive understanding time-frequency distributions are in the following also referred to as “waves”.

[2] Porges SW. The polyvagal perspective. Biological psychology. 2007;74(2):116–143.

[3] Critchley HD, Mathias CJ, Josephs O, O'Doherty J, Zanini S, Dewar BK, et al. Human cingulate cortex and autonomic control: converging neuroimaging and clinical evidence. Brain: A Journal of Neurology. 2003 Oct;126(Pt 10):2139–2152.

[4] Critchley HD, Rotshtein P, Nagai Y, O'Doherty J, Mathias CJ, Dolan RJ. Activity in the human brain predicting differential heart rate responses to emotional facial expressions. NeuroImage. 2005 Feb;24(3):751–762. Available from: http://www.sciencedirect.com/science/article/B6WNP-4DW35VJ-J/2/7ce360f24d5295838b1434f28b95ba55.

[5] Seong H, Lee J, Shin T, Kim W, Yoon Y. The analysis of mental stress using time-frequency distribution of heart rate variability signal. In: Engineering in Medicine and Biology Society, 2004. IEMBS '04. 26th Annual International Conference of the IEEE. vol. 1; 2004. p. 283–285.

[6] Spyers-Ashby JM, Bain PG, Roberts SJ. A comparison of fast fourier transform (FFT) and autoregressive (AR) spectral estimation techniques for the analysis of tremor data. Journal of Neuroscience Methods. 1998 Aug;83(1):35–43.
